# Can the agricultural AquaCrop model simulate water use and yield of a poplar short‐rotation coppice?

**DOI:** 10.1111/gcbb.12422

**Published:** 2017-02-16

**Authors:** Joanna A. Horemans, Hanne Van Gaelen, Dirk Raes, Terenzio Zenone, Reinhart Ceulemans

**Affiliations:** ^1^ Department of BiologyCentre of Excellence PLECOUniversity of AntwerpUniversiteitsplein 1B‐2610WilrijkBelgium; ^2^ Department of Earth and Environmental ScienceK.U.Leuven UniversityCelestijnenlaan 200EB‐3001LeuvenBelgium

**Keywords:** bioenergy, harvestable biomass prediction, POPFULL, *Populus*, soil water content, yield gap

## Abstract

We calibrated and evaluated the agricultural model AquaCrop for the simulation of water use and yield of a short‐rotation coppice (SRC) plantation with poplar (*Populus*) in East Flanders (Belgium) during the second and the third rotation (first 2 years only). Differences in crop development and growth during the course of the rotations were taken into account during the model calibration. Overall, the AquaCrop model showed good performance for the daily simulation of soil water content (*R* ^2^ of 0.57–0.85), of green canopy cover (*R* ^2^ > 0.87), of evapotranspiration (ET; *R* ^2^ > 0.76), and of potential yield. The simulated, total yearly water use of the SRC ranged between 55% and 85% of the water use of a reference grass ecosystem calculated under the same environmental conditions. Crop transpiration was between 67% and 93% of total ET, with lower percentages in the first than in the second year of each rotation. The observed (dry mass) yield ranged from 6.61 to 14.76 Mg ha^−1^ yr^−1^. A yield gap of around 30% was observed between the second and the third rotation, as well as between simulated and observed yield during the third rotation. This could possibly be explained by the expansion of the understory (weed) layer; the relative cover of understory weeds was 22% in the third year of the third rotation. The agricultural AquaCrop model simulated total water use and potential yield of the operational SRC in a reliable way. As the plantation was extensively managed, potential effects of irrigation and/or fertilization on ET and on yield were not considered in this study.

## Introduction

Short‐rotation woody crops (SRCs, cfr. abbreviations in Table 1) with fast‐growing trees are a very promising option for the generation of renewable bioenergy (AEBIOM, 2012). They have high energy‐use efficiency (Boehmel *et al*., 2008) and can mitigate greenhouse gas emissions (Adler *et al*., 2007; Don *et al*., 2011; Njakou Djomo *et al*., 2011; Njakou Domo *et al*., 2013). In comparison with other candidate energy crops, perennial lignocellulosic crops promote biodiversity in an agricultural landscape (Verheyen *et al*., 2014), enhance soil organic carbon storage (Baum *et al*., 2009; Don *et al*., 2011; Berhongaray *et al*., 2016), and improve groundwater quality (Dimitriou *et al*., 2009). In the temperate zones of Europe, poplar (*Populus*) is the most suitable genus for SRC plantations (Dillen *et al*., 2010), but several studies attributed high water consumption to poplar (Zsuffa *et al*., 1996; Meiresonne *et al*., 1999). Some of these studies suggested potential reductions of water table levels and aquifer recharge when extensive SRCs with poplar are established in agricultural areas (Allen *et al*., 1999; Perry *et al*., 2001; Hall, 2003; Petzold *et al*., 2010). These observations have been contradicted by a number of more recent studies (Fischer *et al*., 2013, 2015; Schmidt‐Walter *et al*., 2014; Zenone *et al*., 2015), which reported that evapotranspiration (ET) of poplar SRCs did not exceed the reference ET of a well‐watered grassland (ET_0_) under identical climatological conditions. Obviously, the accurate quantification of the water consumption remains a crucial aspect for the development of poplar SRC and for the conversion of agricultural land into these plantations.

**Table 1:** Description of abbreviations, symbols, and variables used in this contribution

Variable	Description	Units
AGB	Aboveground biomass production	Mg ha^−1^ yr^−1^
*B*	Total (above‐ and belowground) biomass production	Mg ha^−1^ yr^−1^
BGB	Belowground biomass production	Mg ha^−1^ yr^−1^
CC	Green canopy cover	%
CDs	Calender days	
EC	Eddy covariance	
*E*_soil_	Soil evaporation	mm
*E*_soil,tot_	Total annual soil evaporation	mm
ET_0_	Reference crop evapotranspiration	mm
ET_0,tot_	Total annual reference crop evapotranspiration	mm
ET	Evapotranspiration	mm
ET_diff_	Difference of ET between two successive days	mm
ET_tot_	Total annual evapotranspiration	mm
*G*	Soil heat flux	W m^−2^
GDDs	Growing degree days	
*H*	Sensible heat flux	W m^−2^
LAI	Leaf area index	dimensionless
LE	Latent heat flux	W m^−2^
MAD	Mean absolute deviation	
NRMSE	Normalized root‐mean‐square error	%
PAR_i_	Incoming photosynthetically active radiation	W m^−2^
PAR_t_	Transmitted photosynthetically active radiation	W m^−2^
Pr	Precipitation	mm
Pr_tot_	Total annual precipitation	mm
*R* ^2^	Coefficient of determination	
RC	Relative leaf cover of weeds	%
RH	Relative humidity	%
RME	Random measurement error	
*R*_n_	Net radiation	W m^−2^
*R*_s_	Short‐wave radiation	W m^−2^
SRC	Short‐rotation woody crop	
SWC	Soil water content	mm
SWT	Soil water table depth	m
*T*_air_	Average air temperature	°C
*T*_max_	Maximum air temperature	°C
*T*_min_	Minimum air temperature	°C
Tr	Transpiration (component of evapotranspiration)	mm
Tr_tot_	Total annual transpiration	mm
*u*	Wind speed	m s^−1^
*Y*	Yield production = harvestable part of *B*	Mg ha^−1^ yr^−1^

There is a similar need to accurately simulate biomass production and the corresponding energy which can be generated under different climate conditions, site characteristics and/or management options (Headlee *et al*., 2013). For an efficient assessment of the growth, productivity, and water consumption of SRCs, one can rely on various models, ranging from purely empirical models (Ayllot *et al*., 2008) to process‐based forest models (Ceulemans, 1996; Deckmyn *et al*., 2004; Kollas *et al*., 2009; Hart *et al*., 2015). The latter allow to make predictions under the future climate conditions and for different sites (Matala *et al*., 2003) as well as under different management regimes (Korzukhin *et al*., 1996). These models are, however, often complex and parameter rich. Consequently, they require a vast amount of data for input and parameterization that are not always available (Sands *et al*., 2000; Larocque *et al*., 2014). Therefore, the direct use of these models by the end‐users – including land use managers, stakeholders of the bioenergy industry, and policy makers – is often restricted (Mohren & Burkhart, 1994; Matala *et al*., 2003). As SRCs are intensively managed, crop models could be a valuable alternative for forest models to simulate growth, water use, and productivity of SRC. However, in contrast to annual (agricultural) crops, the effect of coppice must be taken into consideration, as well as the recurrent changes in the crop development and in the structure of the SRC within each rotation (Deckmyn *et al*., 2004; Broeckx *et al*., 2015; Zenone *et al*., 2015) and the diversity of poplar species and genotypes (Ceulemans & Deraedt, 1999; Tallis *et al*., 2013).

The AquaCrop model is a simplified process‐based model that simulates soil water content (SWC), crop water use, crop growth, total biomass production (*B*), and yield (*Y*) under different climatological and environmental conditions as well as under different management practices (Raes *et al*., 2009; Steduto *et al*., 2009). In AquaCrop, the biophysical processes are simplified so that the amount of data needed for input and for calibration remains limited, while robustness and accuracy are safeguarded (Vanuytrecht *et al*., 2014b). AquaCrop was initially developed for herbaceous food crops, although forage/feed crops are currently being considered (Kim & Kaluarachchi, 2015). Attempts to use AquaCrop for woody crops are limited to a simulation study of crop transpiration (Tr) of jatropha (Segerstedt & Bobert, 2013) and a study using the water stress function for olive trees (Rallo *et al*., 2012). Furthermore, good results were obtained for the estimation of leaf biomass in tea plantations (Elbehri *et al*., 2015). Our first modelling attempts with AquaCrop for a single‐stem poplar SRC were previously published (Bloemen *et al*., 2016).

In view of the need of user‐friendly tools for water use and yield prediction of SRCs, this study aimed (i) to evaluate the potential of the agricultural model AquaCrop to simulate both water use and harvestable biomass production of SRCs and (ii) to explore the need for further development of the AquaCrop model for the modelling of perennial woody crops. The stand‐level ET, the crop development, and the yield, measured on an operational poplar SRC plantation in East Flanders, Belgium, were used for model evaluation.

## Materials and methods

### Layout and management of experimental plantation

**Figure 1:**
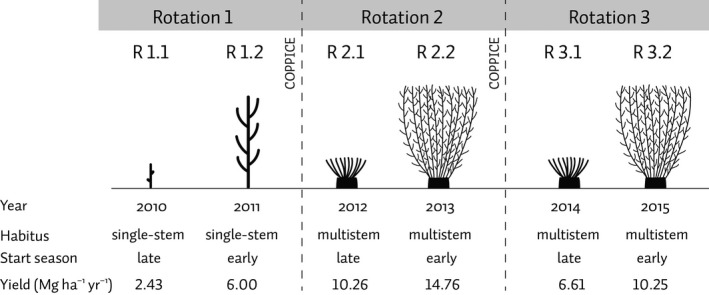
Schematic representation of the crop structure during the three rotations of the short‐rotation poplar plantation in East Flanders (Belgium). A short description of each year of the first rotation (2010–2011; R1.1–R1.2) and of the second rotation (2012–2013; R2.1–R2.2) and the first 2 years of the third rotation (2014–2015; R3.1–R3.2), in terms of habitus, start of the growing season and observed yield, is also presented.

The operational poplar SRC plantation, covering an area of 14.5 ha, was located in Lochristi, East Flanders, Belgium (51°06′44″N, 3°51′02″E, 6.25 m a.s.l.). The area is characterized by a temperate climate with mild winters and cool summers, with rainfall uniformly spread throughout the year. Before the establishment of the SRC plantation, the site was partly cultivated with agricultural crops (sugar beet, corn) and partly used as pasture land. Hardwood cuttings from 12 different *Populus* genotypes representing four parentages and interspecific hybrids were planted in April 2010. Cuttings were planted in a double‐row design with a density of 8000 plants ha^−1^, i.e. 1.1 m within the rows and alternating distances of 0.75 and 1.5 m between the rows (Broeckx *et al*., 2012). For the first two rotations, the plantation was coppiced after 2 years each. The third rotation was prolonged to 3 years. The first 2 years after plantation establishment (2010–2011; R1.1–R1.2) were not considered in this study. The study period consisted of the entire second rotation (2012–2013; R2.1–R2.2) and the first 2 years of the third rotation (2014–2015; R3.1–R3.2). Both rotations were characterized by multistem stumps (with an average of 10 stems per stump; Verlinden *et al*., 2015). The crop structure, as well as the observed yield, and some basic properties are presented in Fig. 1.

Neither fertilization nor irrigation was applied during the entire study period. During the first month after planting and after each coppice, conventional manual and chemical SRC weed control (Ledin & Willebrand, 1996) was applied. More information about the design and the management of the plantation has been previously published (Broeckx *et al*., 2012; Verlinden *et al*., 2013).

### Meteorological and evapotranspiration measurements

Meteorological data were recorded at half‐hourly time steps at the plantation. Air temperature (*T*_air_) and relative humidity (RH) were measured by Vaisala probes (model HMP45C; Vaisala, Helsinki, Finland) and wind speed (*u*) with a sonic anemometer (model CSAT3; Campbell Scientific, Logan, UT, USA). Incoming short‐wave radiation (*R*_s_) was monitored with a pyranometer (model CNR1; Kipp & Zonen, Delft, the Netherlands). Daily precipitation (Pr) records were acquired from a nearby meteorological station of the Royal Meteorological Institute in Zelzate (51°10′53″N, 3°48′33″E, 87.19 m a.s.l.).

Ecosystem‐level fluxes of carbon, water, and energy were continuously monitored from an eddy covariance (EC) mast at the plantation. In this study, only water and energy fluxes were considered. High‐frequency (10 Hz) measurements of the three‐dimensional wind speed components were made using a sonic anemometer. Vertical wind velocity was combined with measurements from a closed‐path, fast‐response gas analyser (model LI‐7000; Li‐Cor, Lincoln, NE, USA) to measure the latent heat flux (LE; Bloemen *et al*., 2016). The LE measurements were aggregated to daily LE values and converted to ET. Additionally, sensible heat (*H*) fluxes were derived from vertical wind speed and sonic temperature measurements. The LE and *H* fluxes and momentum were calculated from half‐hourly aggregates of the high‐frequency measurements using the edire software (R. Clement, University of Edinburgh, UK; http://www.geos.ed.ac.uk/abs/research/micromet/EdiRe/). Gap filling of the original EC data was carried out by the Marginal Distribution Sampling method (Reichstein *et al*., 2005; Lasslop *et al*., 2010) using the standard online tool of Fluxnet (http://www.bgc-jena.mpg.de~MDIwork/eddyproc/). More detailed information about the EC measurements at the site has been previously provided (Zona *et al*., 2013; Zenone *et al*., 2015).

### Soil measurements

In March 2010, just before planting, a detailed soil analysis was performed. The particle size distribution indicated a loamy sand soil with on average 85.7% sand and 11.3% clay (Verlinden *et al*., 2013). The volumetric SWC was continuously monitored at 0.2, 0.3, 0.4, 0.6, and 1 m below the soil surface from the start of the plantation (R1.1) until R3.1 and at 0.05, 0.1, 0.2, 0.5, and 1 m below the soil surface for R3.2, using soil moisture probes (TDR model CS616; Campbell Scientific). Soil water table depth (SWT) was monitored using a pressure transducer (model PDCR 1830; Campbell Scientific). The SWC and SWT were measured at five randomly chosen locations in the vicinity of the technical cabin on the plantation. These locations remained the same over the whole study period. The soil water sensors were changed in R3.2 as the site, a former Fluxnet site (https://fluxnet.ornl.gov) used in the POPFULL project (http://uahost.uantwerpen.be/popfull), became an ICOS site and started following the protocols as described by ICOS (https://www.icos-ri.eu). Analysis of soil samples collected in 2010 and 2014 showed that after 4 years the P, K, Ca, and Mg concentrations in the soil had not significantly changed as compared to the plantation year, and the N concentration in the top soil (0.3–0.6 m) had increased (Vanbeveren *et al*., 2016b). Soil heat flux (*G*) was continuously measured by eight heat flux plates in the soil (HFT3; REBS Inc., Seattle, WA, USA) at 6–8 cm depth (Zona *et al*., 2013).

### Assessment of uncertainty on evapotranspiration

The random measurement error (RME) on the aggregated daily ET data was estimated using a method inspired by the 24‐h approach described by Hollinger & Richardson (2005). Consecutive measurement pairs with a difference in ET_0_ larger than 0.6 mm where excluded from the data set, leaving 72.6% of the data, being 1060 data pairs over the 4 years (2012–2015) of the study. For 10 equally sized groups, grouped according to the size of the ET values, the corresponding RME was calculated as the mean absolute deviation (MAD) of the differences in ET (ET_diff_) between successive days. The MAD was used because the distribution of ET_diff_ was not Gaussian (Shapiro–Wilk: 0.87, *P* > 0.0001, Kolmogorov–Smirnov: 0.14, *P* < 0.01, kurtosis: 9.16, and skewness: 0.036), but double exponential (Laplace). Afterwards, a logarithmic function was fitted through the MAD values as a function of the ET (0.0609 × (ln(ET) + 0.209). This function was used to calculate the RME on all daily ET values. RME was calculated with sas statistical software (version 9.1; SAS Institute Inc., Cary, NC, USA). Another source of uncertainty, the energy balance closure of the EC data, was assessed by the calculation of the regression line between the sum of the measured turbulent fluxes *H* + LE and the available energy Rn − *G* (Rn: net radiation; Foken, 2008; Wohlfahrt *et al*., 2010).

### Plant and crop measurements

Crop development was monitored by leaf area index (LAI) measurements using a LAI‐2200 device (LI‐2200; Li‐Cor). Four replicate LAI measurements were taken per genotype and per former land use type, and measurements were repeated 11–15 times over the growing season (Broeckx *et al*., 2015). The LAI measuring device also gave the amount of transmitted (PAR_t_) and incoming photosynthetically active radiation (PAR_i_) below the canopy. No LAI measurements were available for R3.1.

The aboveground biomass (AGB) was determined from destructive sampling of stems after the coppice of R1 (10 stems per genotype) and R2 (eight stems per genotype). The stems were oven‐dried in the laboratory for 10 days at 70 °C. Afterwards, genotype‐specific allometric (quadratic) relations were obtained between the stem biomass and the stem diameter (Verlinden *et al*., 2013, 2015). Subsequently, the allometric relations were used to calculate the AGB of the poplars for the years without coppice (Broeckx *et al*., 2012; Verlinden *et al*., 2015).

Oven‐dried belowground woody biomass (BGB) was determined by the excavation of the root system (up to 0.6 m depth) and the stump for two genotypes (Koster and Skado) after the coppice of R1 (20 trees) and R2 (6 trees). The values were scaled up to the plantation level taking into account the planting density and the mortality. For fine root sampling, sequential soil cores (between 10 and 20 samples depending on the expected intrinsic variability of the fine root biomass) were taken during rotation years R1.2 and R2.1 and their dry biomass was quantified (Berhongaray *et al*., 2013, 2015).

### The AquaCrop model

AquaCrop simulates crop's *B* and *Y* using a four‐step process. In the first step, the green canopy cover (CC), giving the fraction of the soil covered by green canopy, is simulated. The expansion of CC, from the initial CC (CC_0_) to the maximum CC (CC_x_), is described by a type of logistic function determined by the canopy growth coefficient (CGC). AquaCrop parameters and symbols are summarized in Table 2. Canopy senescence at the end of the growing season is described by means of the canopy decline coefficient (CDC). In the second step, Tr is simulated considering ET_0_, a soil water stress coefficient (*K*_s_) and the crop transpiration coefficient ($K_{c_{\mathrm{Tr}}}$) (Eqn (1)). This $K_{c_{\mathrm{Tr},x}}$ is the product of the simulated CC adjusted for micro‐advective effects (CC*) and the maximum crop transpiration coefficient ($K_{c_{\mathrm{Tr},x}}$). On any day *i*:

(1) $${\text{Tr}}_{i}=K_{s_{i}}\cdot K_{c_{\mathrm{Tr},x}}\cdot {\text{CC}}_{i}^{*}\cdot {\text{ET}}_{0_{i}}.$$

**Table 2:** List of all AquaCrop model parameters with their values for the two calibration years, i.e. the first (R2.1) and the second (R2.2) years of the second rotation

Parameter	Description	Unit	R2.1	R2.2
CC_0_	Initial green canopy cover	%	4	6
CC_x_	Maximum green canopy cover	m² m^−2^	0.96	0.96
CDC	Canopy decline coefficient	fraction GDD^−1^	0.004375	0.002302
CGC	Canopy growth coefficient	fraction GDD^−1^	0.003131	0.004525
Cn	Curve number		46	46
Eme	Period from sowing to emergence	GDDs	0	0
evardc	Effect of canopy cover in reducing soil evaporation in late season	%	70	70
HI	Harvest index ((AGB‐leaves)/*B*)	%	68	68
HIGC	Growth coefficient for HI	day^−1^		
HI_length_	Period of harvest index build‐up (% of the growing cycle)	%	50	50
HI_ini_	Initial value for harvest index	%	0.01	
$K_{c_{\mathrm{Tr},x}}$	Coefficient of maximum crop transpiration		0.99	0.99
*K*_s_	Soil water stress coefficient		1	1
*K*_sat_	Saturated hydraulic conductivity	mm day^−1^	1200	1200
$K_{s_{b}}$	Cold stress coefficient		1	1
Mat	Total length of crop cycle from sowing to maturity	GDDs	3151	3236
mul	Reduction of evaporation by mulches during the growing season	%	21	86
mul_a_	Reduction in soil evaporation by mulches after growing season	%	81	81
mul_b_	Reduction in soil evaporation by mulches before growing season	%	63	81
Root	Period from sowing to maximum rooting depth	GDDs	1683	1385
rtexlw	Maximum root water extraction in bottom quarter of root zone	m^3^ m^−3^ soil day^−1^	0.009	0.009
rtexup	Maximum root water extraction in top quarter of root zone	m^3^ m^−3^ soil day^−1^	0.036	0.036
rt_n_	Minimum effective rooting depth	m	0.8	0.8
rt_x_	Maximum effective rooting depth	m	0.8	0.8
Sen	Period from sowing to start senescence	GDDs	2482	1961
SWC_fc_	Soil water content at field capacity	vol%	22	22
SWC_pwp_	Soil water content at wilting point	vol%	10	10
SWC_sat_	Soil water content at saturation	vol%	41	41
*T*_b_	Base temperature for crop development	°C	0	0
*T*_u_	Upper temperature for crop development	°C	25	25
WP	Water productivity normalized for ET_0_ and CO_2_	g m^−2^	10.4	14

GDDs: growing degree days.

Being a water‐driven model, AquaCrop converts Tr directly into *B* by means of the normalized biomass water productivity (WP) and a cold stress coefficient for biomass production ($K_{s_{b}}$) in the third step (Eqn (2)). WP is the *B* produced per unit land area and per unit of water transpired, normalized for atmospheric CO_2_ and for climate.

(2) $$B=\text{WP}\cdot \sum_{i=1}^{n}K_{s_{b_{i}}}\cdot \frac{{\text{Tr}}_{i}}{{\text{ET}}_{0_{i}}},$$

where *n* is the number of sequential days spanning the growing season. Finally, the harvestable fraction of *B*, referred to as *Y*, is determined by means of the harvest index (HI, Eqn (3)). While for annual crops, *B* is referring to the AGB only, ABG + BGB was considered as *B* for the poplars, and HI was calculated as the fraction of the ABG without the leaves over *B* (AGB + BGB):

(3) $$Y_{i}={\text{HI}}_{i}\cdot B_{i}.$$

The increase in HI over time to reach its final value is described by a logistic function over a specified part of the growing season (HI_length_), starting from an initial HI value (HI_ini_ = 0.01) and a HI growth coefficient (HIGC) (Eqn (4)):

(4) $${\text{HI}}_{i}=\frac{{\text{HI}}_{\mathrm{ini}}\cdot \text{HI}}{{\text{HI}}_{\mathrm{ini}}+\left(\text{HI}-{\text{HI}}_{\mathrm{ini}}\right)\cdot \exp^{-(\mathrm{HIGC})t}},$$

where *t* is the time in days.

During this four‐step simulation process, AquaCrop accounts for the effect of various stresses. To account for water stress, AquaCrop determines the SWC in the root zone using a soil water balance that tracks all incoming (rainfall, eventual irrigation, and capillary rise) and outgoing (run‐off, deep percolation, soil evaporation [*E*_soil_], Tr) daily water fluxes. Next to water stress, AquaCrop also considers the effect of temperature, soil salinity, and soil fertility (Van Gaelen *et al*., 2015). A more detailed description of the AquaCrop model calculation procedures and algorithms has been previously provided (Raes *et al*., 2009, 2012).

### Model input

AquaCrop (version 5) was run using meteorological data of daily ET_0_, Pr, minimum temperature (*T*_min_), and maximum temperature (*T*_max_). Daily ET_0_ values were calculated with the FAO Penman–Monteith equation (Allen *et al*., 1998) based on measured daily *T*_min_ and *T*_max_, minimum and maximum RH, *u* (at 2 m height) and *R*_s_.

Soil parameters (Table 2) were estimated based on the site's soil texture. Default values for a loamy sand soil were used to determine the SWC at field capacity (SWC_fc_) and the SWC at permanent wilting point (SWC_pwp_) as well as for the saturated hydraulic conductivity (*k*_sat_, Raes, 2015). The SWC at saturation (SWC_sat_) was determined based on SWC observations at those days when the soil was completely saturated because of the shallow SWT. Furthermore, the surface run‐off curve number (Cn) was selected based on soil type and forest land use (USDA, 2004), assuming that the initial abstraction equals 5% of surface storage capacity. To consider capillary rise from a shallow SWT, the capillary rise function was calibrated using the default settings for the selected soil texture and *k*_sat_. The SWT over time and the initial SWC were also entered as input in the model.

### Model calibration and evaluation

Crop parameters of AquaCrop were calibrated based on the observations of the second rotation, i.e. the first multistem rotation of the plantation. The calibration took the different growth strategies (Fig. 1) for the two rotation years into account. After calibration, model simulations of daily SWC, ET, and final *Y* were validated against field observations of the third rotation (i.e. first 2 years of R3).

Model evaluation, performed for 2.1, R2.2, R3.1, and R.3.2, was based on the graphs of observed and simulated values as well as on the normalized root‐mean‐square error (NRMSE, normalized over the range of the observed values) and the coefficient of determination (*R* ^2^). Model performance was considered higher when the NRMSE approached 0 or *R* ^2^ values approached 1. The reported *P*‐values refer to overall *F*‐tests, performed to test the hypothesis of a significant linear relationship between observed and simulated values. The model evaluation was carried out, and figures were made with the statistical software r (R 3.2.5, Vienna, Austria. ISBN 3‐900051‐07‐0, URL http://www.R-project.org).

## Results

### Calibration of the model for SRC crop development

The minimum (rt_n_) and maximum (rt_x_) effective rooting depth of the crop was based on values of root length published for the study site (Berhongaray *et al*., 2015), indicating an increasing rooting depth until October of R1.2 and a stable value in R2.1. Therefore, a constant rt_x_ of 0.8 m was chosen for the simulation period. The timing of crop (leaf) emergence, CC_x_, the onset of canopy senescence, and the end of the season were all based on LAI measurements. The measured LAI values were converted to CC values (Eqn (5)), based on Beer's law of light extinction (Raes, 2015).

(5) CC=1−exp(−k·LAI),

where *k* is the light extinction coefficient and *k* was set to 0.6 for poplar using Eqn (6) (Monsi & Saeki, 1953; Sampson & Allen, 1998; Lunagaria & Shekh, 2006), based on PAR_t_ and PAR_i_ measurements in R3.2. This value corresponded to the values reported for poplar (Gielen *et al*., 2003; Hart *et al*., 2015; Oliver *et al*., 2015), although a few lower values had also been observed (Ceulemans *et al*., 1992).

(6) $$k=\left[\frac{-\ln\left({\text{PAR}}_{t}/{\text{PAR}}_{i}\right)}{\text{LAI}}\right].$$

As stems had to regrow from the coppiced stump, the growing season started later in the first year after the coppice (R2.1) than in the second year of the rotation (R2.2). The timing of crop emergence was 6 April 2012 for R2.1 and 20 March 2013 for R2.2. CC_0_ also differed with year within the rotation, with 4% cover for R2.1 and 6% cover for R2.2. Given the changing growth strategies in different years (Fig. 1), the CGC and the CDC for plant development were adapted accordingly via calibration based on simulated and observed CC values. The parameters of crop development were initially entered in calendar days (CDs) and subsequently converted into growing degree days (GDDs) by the model, calculated as *T*_air_ minus the base temperature for plant development (*T*_b_). The validation was then performed using the model based on GDDs.

Depending on the developmental phase of the poplar crop, leaf litter as well as standing vegetation affected the amount of *E*_soil_. A mulch parameter was included in the model to reduce the evaporation caused by mulches covering the ground. The mulch was composed of organic plant material. Although there was no quantitative measure for the mulching effect, the amount of mulches needed was calibrated by comparing the simulated ET with the observed ET. After coppice, which was carried out in winter (February), only a short woody stump remained of the poplars and the mulching effect was low (Table 2: mul_b_ for R2.1 = 21%). During the R2.1 growing season, the mulching effect increased to 63% (mul for R2.1), and after the growing season, when the fallen leaves were covering the ground, to 86% (mul_a_ for R2.1 = mul_b_ for R2.2). During the R2.2 growing season, the limiting effect on the *E*_soil_ remained high (mul of 83%), to restore to 86% (mul_b_ for R2.2) after the growing season until the next coppice.

### Calibration of the model for crop physiology

The $K_{c_{\mathrm{Tr},x}}$ was calculated using eqn (1) based on measured ET and ET_0_ at mid‐season (days on which LAI was between 3.5 and 5), when ET was almost equal to Tr leading to a mean value of 0.99 (90% CI 0.90–1.07). The HI under nonstressed conditions was 68%, with HI_ini_ equal to 0.01% and HI_length_ to 50%. *T*_b_ was set to 0 °C (Pellis *et al*., 2004), and the upper temperature (*T*_u_) for optimal development was set to 25 °C. *T*_u_ is the upper limit of the optimum temperature range (20–25 °C) for deciduous trees in temperate climate regions (Berry & Björkman, 1980). In modelling studies on SRC optimal temperature, values of 20 and 30 °C were used for, respectively, Canada (Amichev *et al*., 2010) and the USA (Headlee *et al*., 2013). Although poplars differ from agricultural crops with respect to habitus, growing cycle, and productivity, the crop parameter ranges were in general applicable to poplars, although some parameters were redundant. Only WP, fine‐tuned at the end of the calibration process by comparing the simulated and observed *B*, was out of the range expected for crops for the first rotation year (10.4 g m^−2^) and on its lower limit for the second rotation year (14 g m^−2^), suggesting a more efficient water use in the second year of R2.

### Environmental conditions

**Figure 2:**
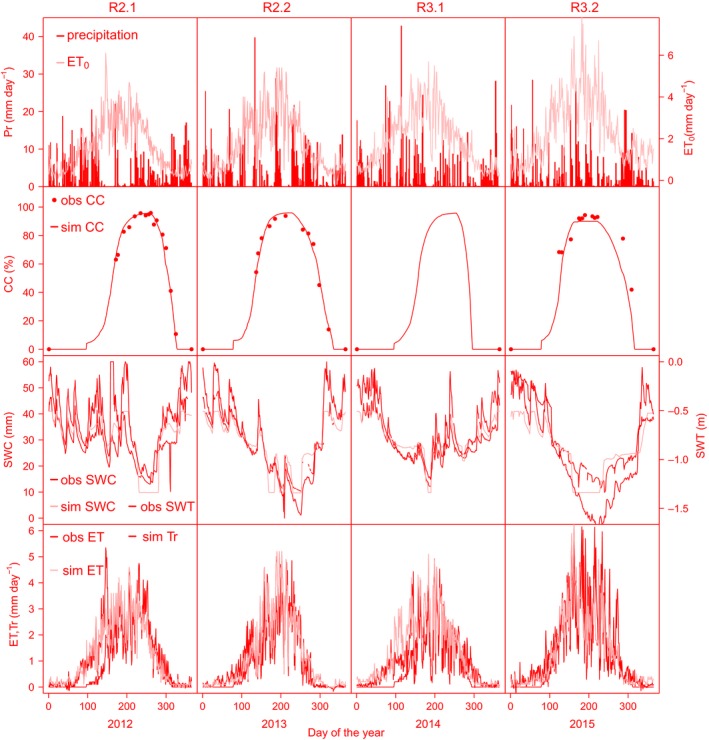
Observed (obs) precipitation (Pr) and calculated reference evapotranspiration of a well‐watered grassland (ET_0_) (top panel row); calculated (from leaf area index (LAI) measurements) and simulated (sim) canopy cover (CC) (second panel row); observed and simulated soil water content (SWC) in the upper 0.3 m of the soil and observed soil water table depth (SWT) (third panel row); and observed and simulated evapotranspiration (ET), and simulated transpiration (Tr) (bottom panel row) for R2 and the first 2 years of R3 of the short‐rotation coppice plantation. For explanations of R2.1, R2.2, R3.1, and R3.2, see Fig. 1.

The variable weather conditions during the two rotations led to highly variable atmospheric water demands, expressed in ET_0_. In particular, in R3.2, some high peaks in water demand, up to 6.2 mm day^−1^, were observed, coinciding with high ET fluxes (Fig. 2). The calculated annual ET_0_ value increased during the study period from 592 mm in R2.1 to 862 mm in R3.2. The annual averages of *T*_min_ and *T*_max_ were also higher in R3 than in R2 (Table 3). The annual Pr totals of the 4 years were very similar and ranged between 789 mm and 852 mm (Table 3). Dryer periods coincided with decreasing SWCs. However, the SWC and SWT were primarily affected by the atmospheric and crop water demand leading to the U‐shaped curves of daily values (Fig. 2) in each year. The SWT was generally quite high and reached the deepest level in R3.2, with a maximum depth of 1.72 m on 12 August 2015.

**Table 3:** Observed and simulated yearly totals of the reference evapotranspiration calculated for a well‐watered grassland (ET_0,tot_), soil evaporation (*E*_soil,tot_), crop transpiration (Tr_tot_) and evapotranspiration (ET_tot_) daily averages, and maxima of evapotranspiration (ET)

Variable	R2.1	R2.2	R3.1	R3.2	Average
Observed					
Yearly					
Pr_tot_ (mm)	788.5	851.3	852.3	804.9	824.3
ET_tot_ (mm)	464.5	372.1	386	536.1	439.7
Daily					
ET average (mm)	1.27	1.02	1.06	1.47	1.21
ET max (mm)	5.4	4.9	4.9	6.2	5.4
Simulated					
Yearly					
ET_0,tot_ (mm)	592	636	713	862	701
E_soil_ (mm)	131	29	163	45	92
Tr_tot_ (mm)	335	368	328	432	366
ET_tot_ (mm)	466	398	491	477	458
ET_tot_/ET_0_	0.85	0.62	0.69	0.55	0.68
Tr_tot_/ET_tot_	0.72	0.93	0.67	0.81	0.78
E_soil,tot_/ET_tot_	0.28	0.07	0.33	0.19	0.22
Daily					
ET average (mm)	1.27	1.17	1.34	1.32	1.28
ET max (mm)	4.6	5.2	5.1	6.5	5.4

For the observations, the total precipitation (Pr_tot_) and the average daily minimum (*T*_min_) and maximum (*T*_max_) temperatures are given. For the simulations, the fractions of ET over ET_0,_ of *E*_soil_ over ET, and of Tr over ET are also shown. For explanations of R2.1, R2.2, R3.1, and R3.2, see Fig. 1.

### Simulations of stand water use

**Figure 3:**
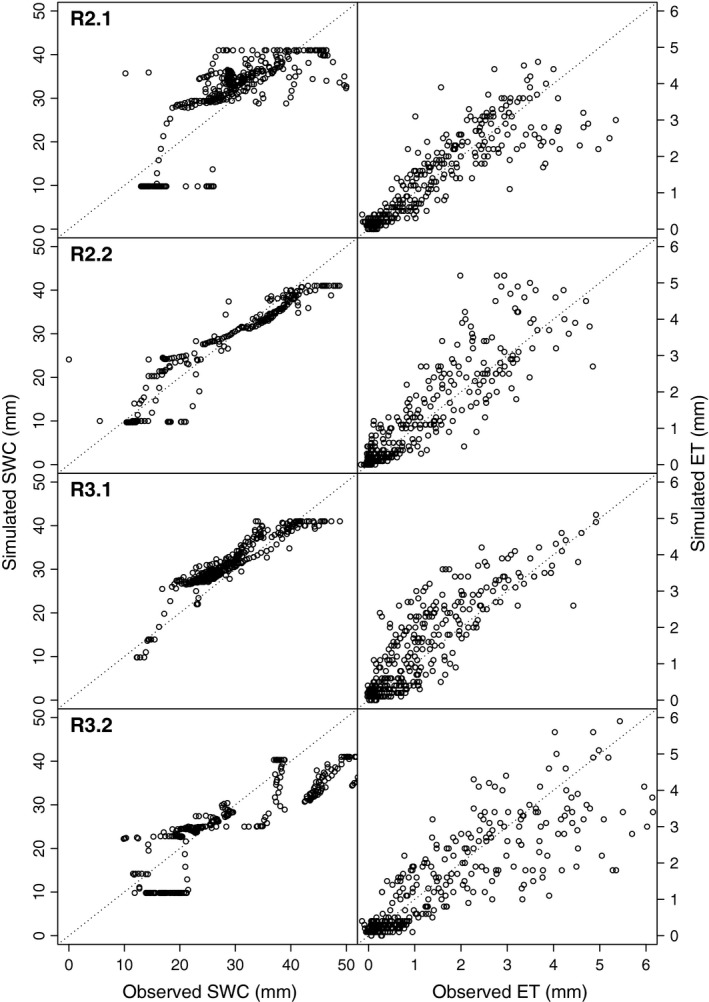
Simulated values of the daily soil water content (SWC) and evapotranspiration (ET) against observed values for all years. For explanations of R2.1, R2.2, R3.1, and R3.2, see Fig. 1. Grey, dotted lines show the 1 : 1 line.

Overall, the AquaCrop model showed good performance for the simulation of SWC and of ET (Figs 2 and 3). The largest deviation of simulated SWC from observed SWC occurred during the second half of R2.1 and the first months of R3.2, where SWC was underestimated. The agreements between simulated and observed were excellent in R2.2 and R3.1 with low relative errors (NMRSE values < 10%, *R* ^2^ = 0.85, *P* < 0.0001, Table 4), but also the model fits for R2.1 and R3.2 captured a significant part of the total variability in the observed SWC (NRMSE = 16.1 and 17, *R* ^2^ = 0.57 and 0.76, respectively, *P* < 0.0001). The strongest deviations from the observations occurred for very low and very high SWC observations.

**Table 4:** Number of observations (*n*), R‐square (*R* ^2^), and normalized root‐mean‐square error (NRMSE, in %) values for the daily soil water content (SWC) in the upper 0.3 m of the soil, for daily evapotranspiration (ET), and for canopy cover (CC) simulations. For explanations of R2.1, R2.2, R3.1, and R3.2, see Fig. 1

Variable	Statistic	R2.1	R2.2	R3.1	R3.2
SWC	*n*	355	231	351	360
	*R* ^2^	0.57	0.85	0.85	0.76
	NRMSE	16.1	9.4	8.1	17.0
ET	*n*	365	365	365	365
	*R* ^2^	0.78	0.81	0.76	0.76
	NRMSE	10.8	10.9	13.6	12
CC	*n*	15	11		11
	*R* ^2^	0.97	0.97		0.87
	NRMSE	5.0	4.6		12.9

**Figure 4:**
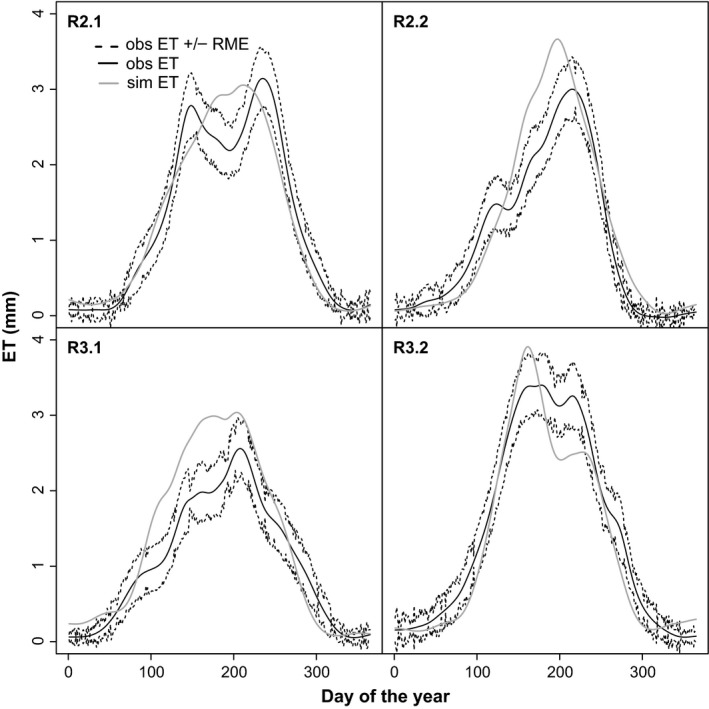
Smoothed spline curves of observed (obs) daily evapotranspiration (ET) values with the (unsmoothed) daily random measurement error (RME), and the smoothed simulated (sim) daily ET values for the 2 years of R2 and the first 2 years of R3. For explanations of R2.1, R2.2, R3.1 and R3.2, see Fig. 1.

The observed ET was significantly well fitted by the model (R^2^ between 0.76 and 0.81, *P* always <0.0001), with NRMSE values between 10% and 15% for all years. However, the simulated ET seemed to miss extreme peaks in observed ET values. The latter was especially true at the onset of the growing season R2.1, and from June till the end of the season in R3.2. The smoothed splines of simulated and observed daily ET values showed that the maximum ET was overestimated in all years and that, for some years, ET raised too fast in the beginning of the season. The simulation results therefore often exceeded the random uncertainty boundaries of the EC measurements between May and August (Fig. 4). Longer periods of overestimated ET values were observed for R2.2 and R3.1. In R3.2, there was a steep decline in simulated ET in June, when SWT started to drop, which caused an underestimation of ET during the summer.

The total annual ET (ET_tot_) of the SRC was lower than the total annual ET_0_ (ET_0,tot_), especially in the second year of both rotations (Table 3) where the fractions of simulated ET_tot_ over ET_0,tot_ were only 0.62 (for R2.2) and 0.55 (for R3.2). For the first year of both rotations, these fractions were higher with values of 0.85 (R2.1) and 0.69 (R3.1). The fraction of the simulated yearly Tr (Tr_tot_) over the simulated ET_tot_ was higher in the second years (>0.8) than in the first years of both rotations (0.72 vs. 0.67). Daily averages of simulated ET in the different years were similar. The average of the simulated daily ET over the 4 years was 1.3 mm day^−1^. For the observed ET, this average value was 1.2 mm day ^−1^. The maxima of daily ET ranged from 4.6 to 6.5 mm day^−1^ for the simulations and from 4.9 to 6.2 mm day^−1^ for the observations. The overall maximum daily ET averaged over the 4 years of the study, 5.4 mm day^−1^, was identical for the simulations and the observations (Table 3).

**Figure 5:**
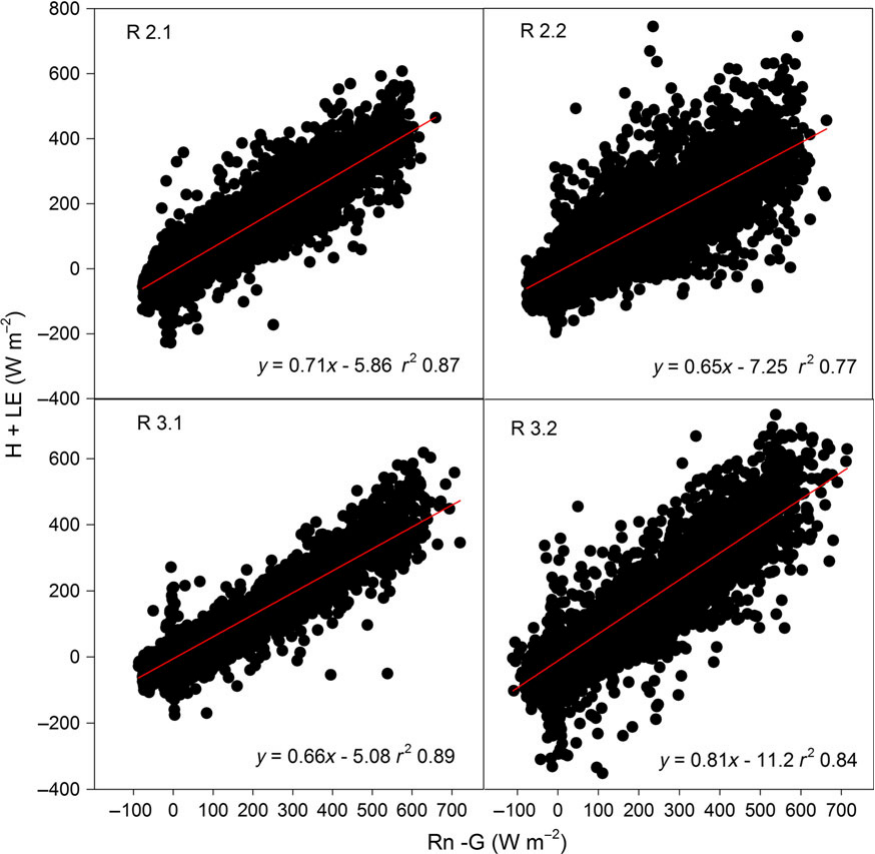
Energy balance closure for the 4 years of the study. Red lines are the linear regression lines. The regression equation between measured energy fluxes and available energy is also presented. For explanations of R2.1, R2.2, R3.1, and R3.2, see Fig. 1.

The energy balance closure at the site ranged from 65% to 81% of the total available energy (Fig. 5) depending on the year and on the time in the growing season. The linear regressions between the available energy and the measured energy fluxes were all highly significant (*P* < 0.0001).

### Simulations of crop development and yield

Relative errors of the CC fits were low (NRMSE < 10%), and the fit was highly significant for the calibration years (*R* ^2^ = 97%, *P* < 0.0001 for both calibration years) and for the validation year R3.2. (NRMSE = 12.9%; *R* ^2^ = 93%, *P* < 0.0001; Table 4). Crop development differed depending on the year with an earlier start of the leaf area development in the noncoppice years (Broeckx *et al*., 2015; Vanbeveren *et al*., 2016a). In the model, the growing season started 18 CDs (or 298 GDDs) later for the first year than for the second year of each rotation, but in both rotation years, the same CC_x_ of 96% was reached. The CC increased somewhat faster when no coppice was executed during the winter before. The total length of the growing season counted 3151 GDDs for R2.1 and 3236 GDDs for R2.2 (Table 2).

The observed (calculated from the measured AGB and BGB) and simulated *B* were, respectively, 21.73 and 22.12 Mg ha^−1^ yr^−1^ for R2.1 vs. 15.11 and 15.01 Mg ha^−1^ yr^−1^ for R2.2. The observed *Y* was markedly higher for the calibration period (R2) than for the validation period (R3), with a decrease of 35.6% for R3.1 compared to R2.1, and of 30.6% for R3.2 compared to R2.2 (Fig. 1, Table 5). *Y* was simulated well for the calibration years, with less than 1% discrepancy. In contrast, *Y* was overestimated for both validation years, with deviations from the observed values of 32.2% for R3.1 and of 33.6% for R3.2 (Table 5).

**Table 5:** Observed vs. simulated yield (Mg ha^−1^ yr^−1^), together with the deviation of the simulated values from the observed values

Year	Observed yield	Simulated yield	Deviation	Relative deviation (%)
R2.1	10.26	10.20	−0.06	−0.58
R2.2	14.76	14.63	−0.13	−0.88
R3.1	6.61	8.74	2.13	32.22
R3.2	10.25	13.69	3.44	33.56

Negative deviation: underestimation; positive deviation: overestimation of the yield. For explanations of R2.1, R2.2, R3.1, and R3.2, see Fig. 1.

## Discussion

In this study, the potential of the AquaCrop model to simulate the water use and the *Y* of SRCs was examined. Based on data from the second and the third rotation of a rainfed poplar SRC, the results evidenced the accurate simulation of ET and Tr by AquaCrop as well as the model's capacity to predict the potential *Y* of SRCs. The yield gap could, at least partly, be explained by the occurrence of an extensive weedy understory layer.

### Simulations of stand water use

The observed and simulated ET_tot_ values were comparable to the ET_tot_ values between 241 and 520 mm reported for extensively managed SRC plantations in Europe (a.o. Schmidt‐Walter *et al*., 2014; Zenone *et al*., 2015; Bloemen *et al*., 2016). The $K_{c_{\mathrm{Tr},x}}$ value for the calibration period (0.99) was compared well with the values published for the Czech Republic (0.92; Fischer *et al*., 2013) and for Germany (0.94; Schmidt‐Walter *et al*., 2014), and was within the range of the values of a comparative literature survey (cfr. table 5 in Fischer *et al*., 2013). Much higher ET_tot_ and $K_{c_{\mathrm{Tr},x}}$ values were reported for well‐irrigated sites with values up to 1100 mm for ET_tot_ and of 4.28 for $K_{c_{\mathrm{Tr},x}}$ in combination with fertilization (Deckmyn *et al*., 2004; Guidi *et al*., 2008; Pistocchi *et al*., 2009). However, high water demands have also been measured on poplars for some unirrigated sites in Europe (Allen *et al*., 1999; Meiresonne *et al*., 1999; Hall, 2003).

Our Tr_tot_/ET_tot_ values were in line with the average value of 0.75 published in a review article using 271 studies on nonfood woody tree plantations (King *et al*., 2013). The fractions were lower for the second years of each rotation when the soil was covered by more standing biomass in the beginning and by more mulch after the growing season (i.e. standing woody biomass and litter). In the beginning of the growing season, more leaf area was transpiring and the resistance for *E*_soil_ was higher. The yearly averages of simulated Tr values were higher than the values from 0.36 to 0.88 mm day^−1^ simulated for the UK with the JULES land‐surface model (Oliver *et al*., 2015). The logarithmic relation between CC and LAI could possibly cause an overestimation of Tr at medium LAI values, when CC had reached its maximum. Another possible difficulty in the simulation of Tr during the growing season is the changing surface conductance of the crop during canopy development (Zenone *et al*., 2015). Finally, the radiation regime and cooler climatic conditions might also explain our higher Tr values.

Difficulties inherent to the use of the SWC sensors might have been the cause of the unrealistically high values of SWC in the original data set. At the start of R3.2, the SWC and SWT sensors were changed, resulting in a strong but temporary bias in that year. One reason for the dissimilarities in the SWC simulations at high observed SWCs is that we limited the SWC to a value of 41% during model calibration.

### Simulations of canopy development and yield

AquaCrop was able to simulate the effect of coppice on the canopy development using two sets of parameters, one for each year of the rotation cycle. The second growing season of the rotation was set to start later compared to the first, as was derived from the LAI data, but also shown by the use of Webcam and MODIS data on the same plantation during R1.2 and R2.1 (Vanbeveren *et al*., 2016a). Developments of the aquacrop software, as to deal with changing crop characteristics of perennial crops, are in progress.

Our observed and simulated *Y* values were well within the range observed and simulated for SRC plantations in the UK (between 5.1 and 16.2 Mg ha^−1^ yr^−1^; Tallis *et al*., 2013; Oliver *et al*., 2015) and in north‐central USA (between 4.4 and 13 Mg ha^−1^ yr^−1^; Headlee *et al*., 2013).

The overestimation of *Y* in both validation years suggests a yield gap in R3. One possible explanation for the 30% yield gap could be the high relative leaf cover of weeds (RC) in R3. The weed infestation became larger in each subsequent rotation and decreased because of light competition over the years within each rotation (direct observations by field technicians), reaching an average RC value of 22% of the total vegetation in R3.3 (assessed by careful inspection of each genotypic block by seven persons on 15 July 2016). For R3.1 and R3.2, the RC was thus larger than 22% and larger than the RC in R2. Weeds grew up to 1.5 m height and accumulated up to 300 g C m^−2^ in biomass (Berhongaray & Ceulemans, 2015; Berhongaray *et al*., 2015) and the weed root biomass reached more than two times the fine root biomass of the poplars already in the first rotation (Berhongaray *et al*., 2013). A technical release of a future version of the AquaCrop model including a weed module (Van Gaelen *et al*., 2016) resulted in a correct estimation of the yield (10.22 Mg ha^−1^ yr^−1^) in R3.2 when an RC of 27% was used. Weakening vitality might also be an explanation for the yield gap (Geyer, 2006). At our site, 15% cutting mortality was observed after R1.1, i.e. during the establishment year (Verlinden *et al*., 2015). Thereafter, mortality remained nearly unchanged until the end of R2 and increased by 5% in R3 (Stefan Vanbeveren, nonpublished data). Nutrient availability did not decrease since the start of the plantation in 2010 (Vanbeveren *et al*., 2016b). However, management (irrigation, rotation length, fertilization, herbicide and pesticide application, harvesting, clone selection) and nutrient availability have interactive effects on the *Y* of SRC plantations (Sabatti *et al*., [Link]). The rather low WP values compared to annual crops (Raes *et al*., 2012) might be due to the relatively high energy cost for lignin production, and hence the enhanced respiration for the wood formation.

### Uncertainties

The model output was restricted by the uncertainties on both the observed and the simulated data. For example, ET observations from EC measurements rely on assumptions during their calculation (Rebmann *et al*., 2012). Furthermore, the lack of energy balance closure in the EC data could cause an underestimation of the ET values (Zona *et al*., 2013; Zenone *et al*., 2015). The model simulations were also prone to uncertainty concerning robustness of the parameters and model structure. Although the simplified and water‐driven structure of the model was effective, the multilayered vegetation structure of perennial trees, even if intensively managed, is more complicated than that of an annual crop. In the process‐based radiation or leaf area‐driven yield models, LAI is a crucial variable (Martin & Jokela, 2004; Fabrika & Pretzsch, 2013; Waring *et al*., 2016). When using the AquaCrop model, data of CC on experimental sites are required to check the influence of the LAI to CC conversion on the simulated ET and consequently also on *Y*. Different techniques, e.g. line intersect sampling, spherical densiometer and digital or satellite imaging, are available to measure the canopy development (Korhonen *et al*., 2006; Vanbeveren *et al*., 2016a). The uncertainties on the model simulations were not considered in this study, but global (Vanuytrecht *et al*., 2014a) and specific (one by one for the most influential parameters) sensitivity analyses (Bloemen *et al*., 2016) of AquaCrop were already performed.

In conclusion, this study demonstrated that the agricultural AquaCrop model enabled to simulate canopy water consumption of a fast‐growing SRC crop in a very reliable way, even if the model's concepts and calculation procedures were originally tailored to annual crops. The model also simulated the gap between potential and realized yield caused by problems in the actual management, by the environment or by dominating RC amounts.
